# Perspectives of Older Adults on Aging Well: A Focus Group Study

**DOI:** 10.1155/2018/9858252

**Published:** 2018-11-04

**Authors:** Hadeel Halaweh, Synneve Dahlin-Ivanoff, Ulla Svantesson, Carin Willén

**Affiliations:** ^1^Department of Health and Rehabilitation, Institute of Neuroscience and Physiology, University of Gothenburg, Gothenburg, Sweden; ^2^Department of Physiotherapy & Rehabilitation, Faculty of Health Professions, Al-Quds University, Jerusalem, State of Palestine; ^3^Centre for Ageing and Health, AGECAP, University of Gothenburg, Gothenburg, Sweden

## Abstract

**Background:**

With increasing number of older adults worldwide, promoting health and well-being becomes a priority for aging well. Well-being and physical and mental health are closely related, and this relation may become more vital at older ages as it may contribute to aging well. The state of well-being is a multifaceted phenomenon that refers to an individual's subjective feelings, and exploring perspectives of older adults on aging well is developing to be an important area of research. Therefore, the aim of this study was to explore perceptions on aging well among older adult Palestinians ≥60 years.

**Methods:**

A qualitative research design in the context of focus group discussions was used; seven focus groups were conducted including fifty-six participants (aged 63–81 years). Data were analyzed using a qualitative interpretative thematic approach described by Braun and Clarke.

**Results:**

Three major themes were identified, *“sense of well-being*,*”* “*having good physical health*,*”* and “*preserving good mental health.”* The participants perceived that aging well is influenced by positive feelings such as being joyous, staying independent, having a life purpose, self-possessed contentment, and financially secured, in addition to be socially engaged and enjoying good physical and mental health.

**Conclusion:**

This study contributes to get a better insight concerning older adults' perspectives on aging well. Enhancing physically active lifestyle, participation in social and leisure activities, healthy eating habits, having a purpose in life, and being intellectually engaged are all contributing factors to aging well. Vital factors are to be considered in developing strategic health and rehabilitative plans for promoting aging well among older adults.

## 1. Introduction

Worldwide, the age group of sixty years old and older is growing faster than any other age group [1]. With this remarkable increase of older adults; promoting health and well-being becomes a priority for aging well [2]. Aging well is conceptualized using different contemporary theoretical frameworks in the last decades, including healthy aging, positive aging, productive aging, active aging, and successful aging [3, 4]. These theoretical frameworks integrate both biological and social sciences, considering social participation, psychology, lifestyles, activities, finances, and other domestic and environmental factors as well [4]. The WHO defines *active aging* [1] *as* “the process of optimizing opportunities for health, participation, and security in order to enhance quality of life as people age including those who are frail, disabled, and in need of care.” As a policy framework [1, 5], active aging allows people to realize their potential for physical, social, and mental well-being throughout the life course and to participate in society. The concept of active aging can be applied for promoting aging well in both developed and developing countries and this is consistent with our study setting in the West Bank/Palestine.

The state of well-being is a multifaceted phenomenon in the older population which generally involves happiness, self-contentment, satisfying social relationships, and autonomy [6]. The sense of well-being refers to an individual's feelings, in this case, based on how older persons perceive the concept of well-being. Thus, the term “subjective well-being” is frequently used [7]. Well-being is also subject to other persons' feelings about oneself whether that is positive or negative. According to McNulty et al. [8], well-being is determined jointly by the interplay between individual characteristics and qualities of people's social environments.

With advanced age, well-being might be adversely influenced by declining physical health and functioning due to age-related changes [9, 10]; older adults may consequently encounter more challenges in pursuing aging well [7, 11, 12]. Maintaining good physical health and functioning plays an important role in facilitating mobility and enables older adults to perform more integrated functional tasks which include activities of daily living, fulfillment of social roles, and recreational activities [7, 13]. Evidence suggests that better physical functioning is associated with physical activity, an interaction that is positively reflected on physical and functional well-being [14–16]. In addition, good physical functioning contributes to decrease falls' incidence [17–19] and prevent the negative impact of falling consequences including social isolation and activity restriction in older adults [20, 21].

Older adults are strongly concerned about cognitive health in term**s** of keeping a good memory and prevent cognitive decline. Therefore, cognitive functioning was addressed as a primary contributor to aging well [22, 23]. Good cognitive health is linked to social connectedness, independence, and life activities, and it might be preserved and enhanced by maintaining an intellectually engaged and physically active lifestyle [24, 25]. Furthermore, having positive mental attitudes towards aging and one's capabilities may contribute to healthier mental health, higher level of satisfaction, and lower levels of anxiety and depression among older adults [26].

Well-being and physical and mental health are closely linked and the link may become more important at older ages, a connection that contributes to aging well in terms of life satisfaction, feelings of happiness, having sense of purpose, and meaning in life [27–30]. In the West Bank/Palestine, about one-third of adult Palestinians ≥18 years reported low levels of well-being; this was influenced by different socioeconomic factors including marital status, living standard, and community participation [31]. These contributing factors to well-being can be more prominent among older adult Palestinians, a marginalized growing population age group with a high rate of poverty, unemployment, and chronic diseases that requires further studies and research [32, 33]. To our knowledge, this is the first qualitative study exploring perceptions of older adult Palestinians on aging well. Thus, the aim of this study was to explore perceptions about aging well among older adult Palestinians who were 60 years old and older at the time of the data collection.

## 2. Methods

### 2.1. Study Design

Focus group method was used to acquire data about perceptions of older adults towards aging well. Focus groups are defined as “carefully planned series of discussions designed to obtain perceptions on a defined area of interest in a permissive, nonthreatening environment” [34]. Group interaction is a fundamental part of this method, in which the vital group discussions among the participants produce the data of the studied topic [35, 36].

### 2.2. Participants

A convenient sample was selected from previous related cross sectional studies [16, 19], addressing physical activity and physical functioning among older adults (*n*=176). The inclusion criteria were being community dwelling older adults (aged ≥60 years), living in the West Bank (Palestine), being able to walk with or without walking aids, and having no communication deficits that would make interviewing and discussions impossible.

In order to obtain a broader view of the participants' perceptions about aging well; heterogeneity was taken into consideration [34, 35] through inviting older adults with different age and levels of education. Issues related to heterogeneity and homogeneity within the groups were considered during groups' formation in order to enhance an interactive discussion and to get a broad range of experience that covering a wide scope of the studied topic [34, 35].

All participants were given verbal and written information about the aim of the study and they signed an informed consent form. The participants were ensured confidentiality and informed that participation was voluntary and that they could drop out of the study at any time. The study received ethical approval from the research Ethics Committee of Al-Quds University, Palestine (Ref No: 1/REC/13), which complies with the Declaration of Helsinki.

### 2.3. Procedure

All focus groups were arranged by the first author as a moderator. Focus group sessions were conducted in familiar, comfortable, and accessible settings for the participants and took place in community and physiotherapy centers in the West Bank (Palestine).

The moderator started the group sessions by welcoming the participants and clarifying the purpose of the study. Subsequently, the participants were invited to introduce themselves and were given a chance to ask if they had any inquiry regarding the study.

The moderator clarified that the discussion would be carried out involving the participants themselves as knowledgeable and expert persons of the studied topic and that the moderator would not contribute to answer the questions. The moderator guided the discussion and encouraged all participants to share and ensured giving sufficient time for each participant to express his or her own view; in addition, comprehension probes were used if needed to clarify responses.

The sessions were initiated with a starting question “*How do you describe aging well*,” followed by these questions:

 
*What do you think about older adults' lifestyles in our community?*
 
*How do you think aging well can be achieved? Meaning how aging well can be facilitated?*
 
*What obstacles stand in the way of aging well?*

The focus groups lasted from 90 to 120 minutes; the interviews were digitally audio-recorded. All interviews were transcribed verbatim by the first author and translated from Arabic to English by the first author in collaboration with a bilingual translator.

### 2.4. Data Analysis

The analyzing process was conducted using the interpretative thematic analysis described by Braun and Clarke [37]. The initial phase of the analysis “becoming familiar with the data” was initiated with reading and rereading the transcripts for several times. Repeated reading contributed to get a better understanding and enhanced researchers' familiarity with the data. Following the initial stage, “generating initial codes (coding)” from the data set that had a reoccurring pattern was performed in the second phase. Coding was carried out through systematic way of organizing and gaining meaningful characteristics of data related to the research question. The first author began the process of initial coding; all transcriptions were coded one by one. Research team meetings were held to discuss the generating initial codes; exchanges that helped create an interpretative space for testing the findings and confirming coding analyses. This process was repeated until coding consensus was reached. The software NVivo 10 [38] was used as a helpful tool for analysis. The third phase *“searching for themes”* focused on a broader level of analysis and involved the researchers identifying suitable themes to which codes could be attributed, initial codes pertinent to research question were integrated into themes considering how relationships were formed between codes and potential themes. To visualize and explore trends and relationships in the source data, codes, and themes, a tree mapping was formulated using NVivo wizards. Derived themes were reviewed in phase four of the analysis, through cyclical process that involves back and forth movements between phases of data analysis until consensus was reached on the final themes. Consequently, in phase five, “Defining and naming themes” was completed, through refining existing themes and subthemes that will be presented in the final analysis.

## 3. Results

### 3.1. Descriptive Information

A total of 56 participants were recruited in this study, the mean age was 68.3 ± 4.72, ranged between 63 and 81 years old. The participants were assigned into different groups according to their place of residency and their preferences to participate in groups that consisted of only women, only men, or women and men together in one group. Accordingly, seven focus groups were formulated: four women groups, two men groups, and one group of women and men together. Participants in all seven groups ranged from 5 to 10 participants. The developed groups were homogenous in terms of independence level and place of residency. The majority of the participants (78%) had one or more chronic diseases, and all participants who lived alone (16%) were women. Participants' demographic and clinical characteristics are illustrated in Table 1. The participants' names were changed to preserve the anonymity of the participants.

### 3.2. Perceptions on Aging Well

Three major themes were identified, and twelve interrelated subthemes were derived. These themes and subthemes were elicited from the discussions for all focus groups combined within the scope to which they were supported by the qualitative data. Themes and subthemes are presented in the following sections.

#### 3.2.1. Sense of Well-Being

Sense of well-being was highlighted through discussions in different focus groups as an important attribute to aging well. This theme was categorized into four related subthemes; feeling joyous, self-possessed contentment, satisfying social relationships, and staying independent.

*(1) Feeling joyous* was viewed as a catalyst to go on in life “joy extends life span,” “joy makes you energetic”; in these words, the participants expressed on the importance of being happy. They have connected happiness with living to advanced age, it was important for them to keep sweet flavor to their lives, no matter how difficult their circumstances were.

 
*Nelly (F, 65y)*: *Despite life is full with troubles and blues, I continue.*
 
*Nadia (F, 73y)*: *I try to be happy, means one accepts everything, no matter bad or good.*
 
*Nelly*: *Yes, we need something taking us out of our concerns and make us happy.*
 
*Shafiq (M, 68y)*: *Absolutely right, being happy is very important for us in this age.*

The participants found ways to add joys to their lives through different strategies such as gatherings, spending time with grandchildren, and sharing activities with others. For them, having leisure activities was of great importance in the context of aging well for older adults.

 
*Fatima (F, 70y)*: *We get together from time to time, where we joke and laugh, listening traditional songs or singing together, this encourages us and makes us happy*.
 
*Sara (F, 64y)*: *For me, the sweetest thing I do is playing with grandson*.
 
*Ibrahim (M, 67y)*: *Yes, grandsons are dearest of sons, they refresh my heart*.
 
*Sara*: *I feel energized; I play with them as a young girl*.

*(2) Self-possessed contentment* was apparently viewed as an important concern for older adults. Throughout the participants' discussions, feeling secured and being satisfied were frequently mentioned, self-contentment was manifested in the necessity of having access to needed resources in terms of health services and daily-life requirements. In this context, feeling financially secured contributed to the state of self-contentment and was described as a facilitating mean to manage life pressures for being able to age well:

 
*Zeinab (F, 65y)*: *When you get old and there is no income, you may be in destitution*.
 
*Ribhieh (F, 67y)*: *Financial status plays a big role in our life*.
 
*Zeinab*: *This brings you many worries and occupies your mind*.
 
*Ibrahim (M, 67y)*: *Yes, the fact if one at ease can eat better can dress better, can live better, this reflects on the state of well-being for us as old people*.

*(3) Satisfying social relationships* were viewed as an attribute to aging well at both familial and community levels. Among different groups' discussions, the participants talked about how important it was for them to be accepted and involved in an area of life. They reflected on how being isolated and lonely might be a serious obstacle to aging well. This subtheme was mostly prominent among women:

 
*Nayfeh (F, 66y)*: *I live alone, nobody knocks my door, that's hard*.
 
*Mariam (F, 70y)*: *I live alone too, but my son lives in the first floor (same building). My daughter lives in town, and they are always around, that helps a lot, they do*n*'t let me alone at all*.
 
*Huda (F, 65y)*: *Yes, It's hard to live alone, but I go out, I share in different occasions, social participation is a good motivator for us as we growing old*.

Staying socially active was described in different phrases and was manifested about “having good neighbors and visiting friends,” “highly motivated person towards life,” and “not being dismal.” Participation in community events was described by some participants as a helpful tool for older adults to stay socially active. Additionally, being socially active was connected with community voluntary work. The participants, both men and women commented on how vital for them it was to do voluntary activities, which helped them as older adults to efficiently spend their free time by doing something sensible to serve their own community. The participants reflected on how voluntary work or being involved in charitable work may enhance their state of well-being.

*(4) Staying independent* was viewed as a major characteristic of aging well. Apart from the participants' living status (alone or with family), the importance of being independent was connected with the autonomous status of the older adults. A major concern that was frequently mentioned was not being or becoming a burden to others. Along the interactive discussions in all groups, the participants reflected on the necessity of staying independent in performing their daily life activities including both personal and instrumental activities.

 
*Seham (F. 65y)*: *I am taking care of myself, my health is good, I need to stay healthy and mentally oriented, so I w*on*'t seek anybody help*.
 
*Hannah (F, 81y)*: *I live alone, I have a big house. I do everything by myself; nobody brings me even a glass of water*.

#### 3.2.2. Throughout the Second Theme “Having Good Physical Health”

Throughout the second theme “having good physical health,” the participants have considered maintaining physical health as an important component of aging well, through our analysis, having good physical health was categorized into five related subthemes; staying active, free from debilitated illness, healthy eating habits, fall prevention, and having a good physical appearance.

*(1) Staying active* was connected to aging well, the participants prominently commented on the importance of staying active by keep on moving. The participants viewed staying active as a key factor for good health. Frequently, the participants talked about staying active in terms of walking which was viewed as a useful tool for aging well; walking was the most prevalent mode of physical activity and has been mentioned repeatedly as a routine activity. Staying active was also connected to good physical functioning, helping older adults to maintain good physical functioning, and keeping good health.

 
*Hannah (F, 81y)*: *I like going out even I have pain in my legs, if I stay home, I will be destructed*.
 
*Salwa (F, 66y)*: *Me too, I' am taking care of my health, I walk a lot*.
 
*Zarifeh (F, 67y)*: *You know, I have a brother in law, he is 81 years old, he is still working and going out everywhere by feet*.

Staying active was also revolved around continuing to work, which was described differently by the participants; men talked more about income generating and community work, while women talked about household and charitable work. Despite there were different perspectives on the concept of “continuing to work,” still it was considered as an important attribute for good physical health and for aging well throughout women and men expressions.

In other circumstances, staying active has been linked to gardening. The participants expressed on the importance of doing some gardening in order to stay active and energetic.

 
*Salem (M, 72y)*: *I work in my garden, and I see myself more active than my sons*.
 
*Nelly (F, 65y)*: *Ohh, I love gardening a lot, I spend like two hours caring of my plants, digging around them. Always my plants look good, my whole garden is tidy, and that keeps me active and energetic*.

*(2) Free from debilitated illness*, staying healthy in terms of absence of debilitated diseases was highlighted during group discussions as an important attribute to good physical health and consequently to aging well. The participants described how occurrences of illness may influence their physical health and their daily-life activities.

 
*Ribhieh (F,67y)*: *Illness sometimes over shadow, I try to forget it, but it is dominated*.
 
*Fatima (F, 70y)*: *After rheumatism, I am not able to walk like before, that affected me a lot*.
 
*Ribhieh*: *Yes, it caused me gloominess sometimes*.
 
*Nelly (F, 65y)*: *Ahh, you know, I underwent two surgeries, and I've suffered a lot, but I've challenged every pain and every disease, I try to live my life*.

*(3) Healthy eating habits* were evoked and being discussed in all groups, the participants reflected on this by focusing on the importance of taking healthy foods. Culturally, ideas regarding the Palestinian diet, which consists mainly of olives oil and lots of vegetables, were mentioned frequently. The participants related healthy diet with good physical health and longevity.

Within this subtheme, promoting healthy eating habits related to aging well were addressed. The participants talked about healthy habits to be taken as well as unhealthy eating style to be skipped. In this context, overeating or getting a full stomach was described as a source of disease, a behavior that has to be prevented in order to maintain good health. Unhealthy eating habits like skipping breakfast and excessive use of salt and sugar were viewed as aggravating factors for some disease symptoms.

*(4) Falls prevention* was a persistent topic that has been discussed among the participants as an important contributor to good physical health. For them, it was vital to stay active but constantly they were concerned about falling. They have viewed falling at this age as a devastating problem, thinking about fall consequences both physically and socially. The participants connected their concern of falling with the associated physical decline.

 
*Majida (F, 70y)*: *I prefer using a cane rather than falling down*.
 
*Wardeh (F,77y)*: *Yah, me too, I pay attention to prevent slipping or falling*.
 
*Majida*: *Before I got sick, there was no problem, I did*n*'t never ever catch any handrails*.
 
*Mariam (F, 70y)*: *Yes I see, it is hard to fall and get fracture at this age, healing is not granted*.

*(5) Having a good physical appearance* was viewed as an important trait to age well. The participants talked about how vital it was for them to keep in good physical appearance; this was connected with “keeping good shape,” “having good stature,” and “maintaining external appearance.” Others pointed out that older persons who are aging well “maintaining good external appearance and dressed well.”

#### 3.2.3. Preserving Good Mental Health

The vitality of maintaining good mental health was viewed as an important attribute to aging well. This theme was categorized into three related subthemes: staying alert, having a positive attitude, and modes to keep good mental health.

*(1) Staying alert*: participants valued their state of mental alertness related to being independent, having control over their own affairs, and being self-governing. Being mentally alert and having a good memory was mentioned frequently as an imperative dimension of aging well.

 
*Salma (F, 68y)*: *I was living with my mother in law, she reached her nineties, and she had a clear mind, everyone respected her, and I've learned a lot from her*.
 
*Ribhieh (F, 66y)*: *Yes I agree, If the brain is still good, you will be fine*.

*(2) Having a positive attitude* emerged as an important attribute to aging well, and it was diversely characterized as follows: “my spirit is strong, I just follow my mind,” “I don't let anybody put me down,” and “I'm in any way, I want to live.” The participants commented on the importance of getting this impulse of life by staying positive. In this context, having positive attitudes towards own capabilities as older adults was reflected by fulfilling own ambitions and having a life purpose in pursuit of one's aspiration.

Being positive was also connected with spiritual merits, having faith, praying, and trust in God; these traits have been mentioned frequently through discussions in different groups. The participants commented on the importance of reaching a state of serenity and tranquility as an important attribute to aging well.

*(3) Modes to keep good mental health* including actions such as reading newspapers and books, watching TV and listening to the radio, eating certain foods like nuts, staying active, and playing mental games. All these statements were mentioned to describe taken activities by the participants in order to keep good mental health and to age well:

 
*Farida (F, 64y)*: *I read newspaper every day, that keeps me oriented*.
 
*Nayfeh (F, 66y)*: *For me, I can't read well, watching TV and hearing radio amuses me and keeps me alert*.
 
*Hannah (F, 81y)*: *I work with letters and numbers, Sudoku is good if you manage to deal with it*.
 
*Farida*: *Ohh, That's good too*.

Willingness to learn new skills was also considered by some participants as a way to keep a good mind; the new skills were mostly revolving around computer uses, handcrafts, and simple maintenance work.

## 4. Discussion

Happiness, self-contentment, satisfying social relationships, and independence are primary characteristics of the state of well-being that contribute to aging well in the older population [6]. A study by Tamir and Ford [39] indicated that people who generally wanted to feel more happiness and less anger experienced greater well-being. Corresponding with our results, as illustrated in the first theme, the participants have considered joy and happiness an important tool to age well. Feeling joyous was viewed as a catalyst to go on in life and was connected with living to advanced age. When older adults experience well-being, they are also experiencing the sense of self-contentment which is connected to the feeling of being happy and satisfied. Self-contentment in this study was manifested in feeling financially secured and in the necessity of having access to needed resources in terms of health services and daily-life requirements. The findings are consistent with a similar study [40], where financial security appeared to be an essential contributor to aging well.

Well-being is also subject to how a person feels that other people in their surroundings perceive them, whether this is positive or negative [8]. The findings are corresponding with our results in the subtheme “satisfying social relationships,” and the participants commented on the importance of having social connections at both familial and community levels. These findings are in harmony with the concept of active aging [1], which enables older people to realize their diverse potentials for well-being. However, the well-being of a person does not only depend on the individual, rather well-being has a social component as well, and it is determined jointly by the interplay between individual characteristics and qualities of people's social environments [39].

The participants reflected on how sensible for them it was not to be ignored and isolated at this age, and they thought being isolated and lonely as a serious obstacle to aging well. This concern was mostly prominent among older women who are more likely to spend their later stage of their lives alone [32]. Related to literature, living alone and low social participation were found to be significant risk factors for later disability onset [41]. Older adults who live alone report more fatigue and more health difficulties than older adults who do not live lonely [42, 43], issues that are negatively contributing to aging well.

Our findings revealed that autonomy and independence were viewed as primary attributes to age well. Concern of being a burden to others was very prominent throughout participants' expression. Older adults in other cultural circumstances as well placed a high value on personal independence and self-reliance, where staying independent was viewed as a major trait for aging well [24, 44]. The participants related their level of independence to their physical and mental health; a sensible understandable relation as a higher level of physical functioning enables older adults to perform more integrated functional tasks which include activities of daily living and the fulfillment of social roles as well as recreational activities [7], issues that are essential to age well.

Throughout the second theme “having a good physical health,” the participants connected their good physical health with staying active. Often, they have talked about staying active in terms of walking. This can be explained as walking was viewed as one of the most popular forms of physical activity among older adults and can easily be adapted into daily lifestyle [45].

In this study, walking was connected to good physical functioning and has been mentioned repeatedly as a routine activity, helping older adults to maintain good physical functioning and keeping good health. Findings are consistent with similar studies indicating that walking is positively associated with physical and functional well-being in older adults [14, 15].

Physical health was addressed according to Phelan et al. as being in good health and absence of chronic diseases [46]. In Rowe and Kahn's model, it was addressed as avoiding disease and maintaining high cognitive and physical function [47]. In this study, the majority of the participants, about 78% had one or more chronic diseases. For them, the concern about physical health in term of diseases was more prominently about being free from debilitated illness that may incapacitate their abilities and limit their daily-life activities. Results that are consistent with a related study showed that participants with higher prevalence of chronic diseases recorded lower level of physical activity [15].

Promoting healthy eating habits related to aging well was also addressed in this study. The participants related healthy diet with good physical health and consequently to aging well, a subtheme that was also found among perceptions of older Japanese adults in a study towards aging well [40].

Additionally, falls prevention was a persistent topic that has been discussed among the participants as an important contributor to good physical health. For them, especially women, it was imperative to stay active but constantly they were concerned about falling. This can be attributed to the fact that higher incidence of falls is associated with higher age, and women tend to fall more frequently than men [48, 49]. Participants have viewed falling at this age as a big problem, thinking about fall consequences both physically and socially. A subtheme is consistent with other studies [17–19], which have shown that good physical health and physical functioning play an important role in decreasing falls' incidence and fear of falling and, in turn, prevent the negative impact of falling consequences including social isolation, activity restriction, and enhance state of well-being in older adults [20, 21].

In this study, preserving good mental health and staying alert were viewed as important attributes to aging well, and good mental health was connected to staying independent and being self-governing. In a study by Laditka et al. [24], maintaining good cognitive health was linked to social connectedness, independence, and life activities that are difficult to maintain with poor cognitive health. Within the second theme, “having a positive attitude” was described as an attribute to good mental health and to age well. This can be understandable as having positive attitudes towards aging and owns capabilities may contribute to healthier mental health. More positive attitudes were associated with higher level of satisfaction and lower levels of anxiety and depression in older adults [26].

Within this theme, participants related having a positive attitude to spiritual merits, having faith, praying, and trust in God. These traits have been mentioned frequently in different groups where the participants commented on the importance of reaching a state of serenity and tranquility that can be achieved through spiritual dimension as an important attribute to aging well. A spirituality dimension of aging well was found in similar studies [24, 40] as well under categories such as faith, religion, blessings, and internal peace.

The state of well-being is positively influenced by having a life purpose that can motivate older adults to sustain independence, social life, and make life meaningful for older adults [29]. The feeling of having a purpose in life was also contributing to aging well in our study that was manifested by fulfilling ambitious and having a life goal in pursuit of one's aspiration.

Evidence suggests that cognitive functioning may be preserved and enhanced by maintaining an intellectually engaged and physically active lifestyle. Meaningful social engagement is also an important factor of better maintenance of cognitive functioning in old age [25]. Keeping good mental health was an important concern for older adults in this study; different modes were described to maintain good mental health including being mentally engaged (reading, playing mental games), taking good foods for the brain like nuts and staying physically active.

### 4.1. Strengths and Limitations

The aim of this study was to explore perceptions about aging well among older adults, as the state of aging well refers to an individual's subjective feelings and is basically dependent on the older adults' views [7, 50]. Therefore, a qualitative research design in the context of focus group discussions was used; this qualitative thematic analysis approach contributed to get better insight into older adults' perceptions and experiences that cannot be elicited through quantitative studies.

Using focus group discussion as a method of data collection has enabled the researchers to get both individual and interactive opinions by the participants. This method is effectively used in research on aging [51, 52], and it is considered appropriate for collecting the views and experiences of a selected group through dynamic interaction and vital group discussion of a studied topic [35, 36]. In addition, focus group method was used, because it is a friendly respectful research method and not a condescending method [53] to be used with older adult participants. To assure permissive and nonthreatening environment for conducting this study [34], participants were assigned into different groups according to their place of residency and their preferences to participate in groups that consist of only women, only men, or women and men together. This procedure contributed to a relaxed discussion atmosphere through having familiar, comfortable, and accessible settings for the participants.

The groups' size in this study was determined based on the research question, taking into consideration that small groups with less than five participants may limit the range of interactive discussions, while large groups meaning more than ten participants can be hard to be managed by the moderator and may bound the participants' opportunities to share their thoughts and experiences [34, 54].

The relationship between the researcher (first author) and the participants has been developed progressively through several interviews. Our sample was selected conveniently from previous related cross-sectional studies [16, 19], addressing physical activity, physical functioning, and fall-related efficacy among older adults. Familiarity of researcher with the participants gave a chance to create a comfortable interviewing atmosphere, which helped the researcher build a trusting connection with the participants and encouraged the participants to talk more freely.

This study addresses aging well in a holistic manner that includes state of well-being, physical and mental health, independence, and social participation [3, 55]. This helped us in giving a better understanding about the interaction between different physical, social, and mental functioning dimensions regarding the state of well-being among older adults. However, further studies addressing each dimension in more depth may add additional evidence towards a better understanding of the concept of aging well.

A possible limitation of this study could be that we recruited older adults who are relatively independent and functioning and living in the community at own homes within a family or alone, and most of them rated their self-fitness between good and very good. Further studies are needed to explore perspectives on aging well among older adults living in institutions with lower level of functioning and independence.

## 5. Conclusion

This study gives in-depth understanding of the dynamic multidimensional physical, social, and mental functioning on the state of well-being among older adults. Findings contribute to get better insight about older adults' perspectives on aging well. Aging well is positively influenced by feeling joyous, staying independent, self-possessed contentment, and being financially secured, in addition to being socially engaged and enjoying good physical and mental health. Enhancing a physically active lifestyle, social participation, and leisure activities as well as healthy eating habits and having a purpose in life and intellectually engagement are all important factors to promote aging well. Vital factors are to be considered in developing strategic health and rehabilitative plans for promoting aging well among older adults.

## Figures and Tables

**Table 1:** Demographic and clinical characteristics of the participants (*n*=56).

Variables	All groups	Group 1	Group 2	Group 3	Group 4	Group 5	Group 6	Group 7
	*n*=56	*n*=10	*n*=8	*n*=7	*n*=9	*n*=5	*n*=10	*n*=7
Gender								
** **Women, *n* (%)	40 (71)	10		7	9		7	7
** **Men, *n* (%)	16 (29)		8			5	3	

Marital status								
** **Married, *n* (%)	41(73)	7	8	6	4	5	7	4
** **Single, *n* (%)	3 (5)				2		1	
** **Widowed, *n* (%)	12 (22)	3		1	3		2	3

Living status								
** **With family, *n* (%)	47 (84)	7	8	6	8	5	7	6
** **Alone, *n* (%)	9 (16)	3		1	1		3	1

Diagnosed disease, *n* (%)								
** **Yes	44 (78)	8	6	6	7	4	9	4
** **No	12 (22)	2	2	1	2	1	1	3
** **Hypertension	30 (54)	6	6	3	4	2	7	2
** **Diabetes	14 (25)	4	1	2	3		3	1
** **Musculoskeletal	24 (43)	6		4	5	2	4	3

Use assistive aid, *n* (%)								
** **Glasses	33 (59)	5	4	4	6	4	5	5
** **Walking aid (cane)	5 (9)	1		2		1	1	

Self-rated fitness, *n* (%)								
** **Poor	5 (9)	1		2	1			1
** **Quite good	22 (39)	4	1	3	3	2	7	2
** **Good	22 (39)	5	4	1	4	2	3	3
** **Very good	7 (13)		3	1	1	1		1

## Data Availability

The data used to support the findings of this study are available from the corresponding author upon request.

## References

[1] World Health Organization (2002). *Active Ageing, A Policy Framework*.

[2] World Health Organization (September 2014). Facts about ageing. http://www.who.int/ageing/about/facts/en/.

[3] Foster L., Walker A. (2015). Active and successful aging: a European policy perspective. *Gerontologist*.

[4] Buys L., Miller E. (2012). *Active Ageing: Developing a Quantitative Multidimensional Measure, Active Ageing, Active Learning*.

[5] Kalache A., Gatti A. (2003). Active ageing: a policy framework. *Advances in Gerontology*.

[6] Kunzmann U., Little T. D., Smith J. (2000). Is age-related stability of subjective well-being a paradox? Cross-sectional and longitudinal evidence from the Berlin aging study. *Psychology and Aging*.

[7] Spirduso W. W., Francis K. L., Macrae P. G. (2005). *Physical Dimensions of Aging*.

[8] Mcnulty J. K., Fincham F. D. (2012). Beyond positive psychology? Toward a contextual view of psychological processes and well-being. *American Psychologist*.

[9] Fusco O., Ferrini A., Santoro M., Lo Monaco M. R., Gambassi G., Cesari M. (2012). Physical function and perceived quality of life in older persons. *Aging Clinical and Experimental Research*.

[10] Garatachea N., Molinero O., Martinez-Garcia R., Jimenez-Jimenez R., Gonzalez-Gallego J., Marquez S. (2009). Feelings of well being in elderly people: relationship to physical activity and physical function. *Archives of Gerontology and Geriatrics*.

[11] Steverink N., Lindenberg S. (2006). Which social needs are important for subjective well-being? What happens to them with aging?. *Psychology and Aging*.

[12] Liu Y., Dijst M., Geertman S. (2017). The subjective well-being of older adults in Shanghai: the role of residential environment and individual resources. *Urban Studies*.

[13] Halaweh H., Svantesson U., Willen C. (2016). Experiences of habitual physical activity in maintaining roles and functioning among older adults: a qualitative study. *Rehabilitation Research and Practice*.

[14] Hörder H., Skoog I., Frändin K. (2013). Health-related quality of life in relation to walking habits and fitness: a population-based study of 75-year-olds. *Quality of Life Research*.

[15] Halaweh H., Willen C., Grimby-Ekman A., Svantesson U. (2015). Physical activity and health-related quality of life among community dwelling elderly. *Journal of Clinical Medicine Research*.

[16] Halaweh H., Willén C., Svantesson U. (2017). Association between physical activity and physical functioning in community-dwelling older adults. *European Journal of Physiotherapy*.

[17] Yamashita T., Jeon H., Bailer A. J., Nelson I. M., Mehdizadeh S. (2011). Fall risk factors in community-dwelling elderly who receive Medicaid-supported home- and community-based care services. *Journal of Aging and Health*.

[18] Delbaere K., Van Den Noortgate N., Bourgois J., Vanderstraeten G., Tine W., Cambier D. (2006). The physical performance test as a predictor of frequent fallers: a prospective community-based cohort study. *Clinical Rehabilitation*.

[19] Halaweh H., Willen C., Grimby-Ekman A., Svantesson U. (2015). Physical functioning and fall-related efficacy among community-dwelling elderly people. *European Journal of Physiotherapy*.

[20] Legters K. (2002). Fear of falling. *Physical Therapy*.

[21] Tinetti M. E., Mendes De Leon C. F., Doucette J. T., Baker D. I. (1994). Fear of falling and fall-related efficacy in relationship to functioning among community-living elders. *Journals of Gerontology*.

[22] Fillit H. M., Butler R. N., O’connell A. W. (2002). Achieving and maintaining cognitive vitality with aging. *Mayo Clinic Proceedings*.

[23] Bourassa K. J., Memel M., Woolverton C., Sbarra D. A. (2017). Social participation predicts cognitive functioning in aging adults over time: comparisons with physical health, depression, and physical activity. *Aging and Mental Health*.

[24] Laditka S. B., Corwin S. J., Laditka J. N. (2009). Attitudes about aging well among a diverse group of older americans: implications for promoting cognitive health. *Gerontologist*.

[25] Hertzog C., Kramer A. F., Wilson R. S., Lindenberger U. (2008). Enrichment effects on adult cognitive development: can the functional capacity of older adults be preserved and enhanced?. *Psychological Science in the Public Interest*.

[26] Bryant C., Bei B., Gilson K., Komiti A., Jackson H., Judd F. (2012). The relationship between attitudes to aging and physical and mental health in older adults. *International Psychogeriatrics*.

[27] Ryff C. D. (1995). Psychological well-being in adult life. *Current Directions in Psychological Science*.

[28] Mitchell V., Helson R. M. (2016). The place of purpose in life in women’s positive aging. *Women & Therapy*.

[29] Irving J., Davis S., Collier A. (2017). Aging with purpose: systematic search and review of literature pertaining to older adults and purpose. *International Journal of Aging and Human Development*.

[30] Steptoe A., Deaton A., Stone A. A. (2015). Subjective wellbeing, health and ageing. *The Lancet*.

[31] Harsha N., Ziq L., Ghandour R., Giacaman R. (2016). Well-being and associated factors among adults in the occupied Palestinian territory (oPt). *Health and Quality of Life Outcomes*.

[32] Palestinian Central Bureau of Statistics (December 2017). Dissemination and analysis of census findings: the conditions and requirements of elderly care in the Palestinian territory 1997-2007. http://www.pcbs.gov.ps/Portals/_PCBS/Downloads/book1647.pdf.

[33] Palestinian Central Bureau of Statistics (December 2017). On the eve of world elderly day. http://www.pcbs.gov.ps/post.aspx?lang=en&ItemID=1755.

[34] Krueger R. A., Casey M. A. (2009). *Focus groups: A practical Guide for Applied Research*.

[35] Kitzinger J. (1994). The methodology of focus groups: the importance of interaction between research participants. *Sociology of Health & Illness*.

[36] Ivanoff S. D., Hultberg J. (2006). Understanding the multiple realities of everyday life: basic assumptions in focus-group methodology. *Scandinavian Journal of Occupational Therapy*.

[37] Braun V., Clarke V. (2006). Using thematic analysis in psychology. *Qualitative Research in Psychology*.

[38] Edhlund B. (2012). *NVivo 10 Essentials*.

[39] Tamir M., Ford B. Q. (2012). Should people pursue feelings that feel good or feelings that do good? Emotional preferences and well-being. *Emotion*.

[40] Iwamasa G. Y., Iwasaki M. (2011). A new multidimensional model of successful aging: perceptions of Japanese American older adults. *Journal of Cross-Cultural Gerontology*.

[41] Lund R., Nilsson C. J., Avlund K. (2010). Can the higher risk of disability onset among older people who live alone be alleviated by strong social relations? A longitudinal study of non-disabled men and women. *Age Ageing*.

[42] Deng J. L., Hu J. M., Wu W. L., Dong B. R., Wu H. M. (2010). Subjective well-being, social support, and age-related functioning among the very old in China. *International Journal of Geriatric Psychiatry*.

[43] Gilmour H. (2012). Social participation and the health and well-being of Canadian seniors. *Health Reports*.

[44] Shaw R., Langman M. (2017). Perceptions of being old and the ageing process. *Ageing International*.

[45] Atalay O. T., Cavlak U. (2012). The impact of unsupervised regular walking on health: a sample of Turkish middle-aged and older adults. *European Review of Aging and Physical Activity*.

[46] Phelan E. A., Anderson L. A., Lacroix A. Z., Larson E. B. (2004). Older adults’ views of “successful aging”–how do they compare with researcher’s definitions?. *Journal of the American Geriatrics Society*.

[47] Rowe J. W., Kahn R. L. (1997). Successful aging. *Gerontologist*.

[48] Luk J., Chan T. Y., Chan D. K. (2015). Falls prevention in the elderly: translating evidence into practice. *Hong Kong Medical Journal*.

[49] Bergland A. (2012). Fall risk factors in community-dwelling elderly people. *Norsk Epidemiologi*.

[50] Stewart A. L., King A. C. (1991). Evaluating the efficacy of physical activity for influencing quality-of-life outcomes in older adults. *Annals of Behavioral Medicine*.

[51] Finn V., Ellis R. (2006). Media Review: Curry, L., Shield, R., & Wetle, T. (2006). Improving aging and public health research: qualitative and mixed methods. Washington, DC: American Public Health Association and the Gerontological Society of America. *Journal of Mixed Methods Research*.

[52] Costello E., Kafchinski M., Vrazel J., Sullivan P. (2011). Motivators, barriers, and beliefs regarding physical activity in an older adult population. *Journal of Geriatric Physical Therapy*.

[53] Morgan D. L., Krueger R. A. (1993). *Successful Focus Groups: Advancing the State of the Art*.

[54] Gill P., Stewart K., Treasure E., Chadwick B. (2008). Methods of data collection in qualitative research: interviews and focus groups. *British Dental Journal*.

[55] Foster L., Walker A. (2013). Gender and active ageing in Europe. *European Journal of Ageing*.

[56] Halaweh H. Active ageing a path towards ageing well. physical functioning, physical activity, falls self-efficacy and social participation in community-dwelling elderly.

